# Caregiving Self-Efficacy of the Caregivers of Family Members with Oral Cancer—A Descriptive Study

**DOI:** 10.3390/healthcare11050762

**Published:** 2023-03-05

**Authors:** Ching-Hui Cheng, Shu-Yuan Liang, Ling Lin, Tzu-Ting Chang, Tsae-Jyy Wang, Ying Lin

**Affiliations:** 1 Department of Nursing, Cheng Hsin General Hospital, Taipei 112, Taiwan; 2 School of Nursing, National Taipei University of Nursing and Health Sciences, Taipei 112, Taiwan; 3 Department of Nursing, Taipei Veterans General Hospital, Taipei 112, Taiwan

**Keywords:** caregiving self-efficacy, family caregiver, oral cancer

## Abstract

In Taiwan, oral cancer is the fourth most common cause of cancer death in men. The complications and side effects of oral cancer treatment pose a considerable challenge to family caregivers. The purpose of this study was to analyze the self-efficacy of the primary family caregivers of patients with oral cancer at home. A cross-sectional descriptive research design and convenience recruiting were adopted to facilitate sampling, and 107 patients with oral cancer and their primary family caregivers were recruited. The Caregiver Caregiving Self-Efficacy Scale-Oral Cancer was selected as the main instrument to be used. The primary family caregivers’ mean overall self-efficacy score was 6.87 (SD = 1.65). Among all the dimensions, managing patient-related nutritional issues demonstrated the highest mean score (mean = 7.56, SD = 1.83), followed by exploring and making decisions about patient care (mean = 7.05, SD = 1.92), acquiring resources (mean = 6.89, SD = 1.80), and managing sudden and uncertain patient conditions (mean = 6.17, SD = 2.09). Our results may assist professional medical personnel to focus their educational strategies and caregiver self-efficacy enhancement strategies on the dimensions that scored relatively low.

## 1. Introduction

In 2018, oral cancer incidence and death were the highest among men in Taiwan, and oral cancer was the fourth most common cause of cancer-induced death in men [1]. When patients with oral cancer undergo treatment and experience its side effects [2,3,4], patients themselves, their families, and medical caregivers encounter great challenges in caregiving.

Stage classification of oral cancer includes four stages according to the size of the primary tumor (T), involvement of locoregional lymph nodes (N), and distant metastases (M) [5,6,7,8]. Stage I is determined by T1–2 and N0–1, stage II by T1–2 and N2 or T3 and N0–2, and stage III by T4 or N3. Stage IV is for patients with metastatic disease [7]. This classification can aid in treatment planning, the estimation of recurrence risk, and the assessment of patient survival [5]. The overall 5-year survival rate for patients in a cohort study at Memorial Sloan Kettering Cancer Center was 63% [9]. In a multicenter retrospective analysis, an advanced T stage was significantly correlated with poor overall survival and disease-specific survival of patients [10]. Lymph node involvement is the most important prognostic factor in oral cancer. The survival rate is reduced by 50% when compared with those with similar primary tumors without neck lymph node involvement [11,12]. The impact of oral cancer at different stages on patients’ physical symptoms and impairments was supported, especially the impact of advanced oral cancer [13].

Oral cancer treatment may involve the combined use of surgery, chemotherapy, and radiotherapy, among which surgery is the most essential [14]. However, surgical treatment may change patients’ facial appearance and cause oral disabilities, such as impaired communication and eating functions [2]. In addition, patients with oral cancer encounter the side effects of chemotherapy or radiotherapy. Therefore, care for oral cancer is more challenging than that for other cancers [15].

In Taiwan, family members play a crucial role in the home care of patients with oral cancer, as exemplified by the trends during outpatient treatment. For instance, these family members handle patients’ nutritional problems, make care decisions, manage disease-related emergencies, and seek relevant resources [16]. However, the difficulties they encounter during home care [16] may discourage these family members from putting effort into patient care, particularly when they lack belief in their own capability, worsening the subsequent care results.

Self-efficacy refers to an individual’s capability belief or perceived capability to perform specific health care behavior [17]. During health care processes, self-efficacy is an essential ability that helps individuals overcome difficulties and strive for better health [18]. Self-efficacy is a key factor that affects health care behavior [19] because self-efficacy positively affects individuals’ behavioral motivation and persistence when they encounter care difficulties [18].

In the research literature, investigations that examined the difference in gender regarding self-efficacy produced inconsistent findings. Several researchers described self-efficacy as one factor that accounts for gender differences [20,21]. While some researchers suggested that men reported greater self-efficacy than women [20], others suggested that females reported greater efficacy than men [21]. In contrast, no gender differences regarding self-efficacy ratings were noted in some studies [22,23,24].

Bandura [25] also suggested that age may be a factor that contributes to personal efficacy due to the biological processes of aging resulting in declining ability. Research on the effects of age on self-efficacy has produced mixed results [20,22,23,24]. Several studies indicated no relationship between self-efficacy ratings and age [20,22,24].

Educational and socio-economic levels may also be personal factors that are associated with self-efficacy since they lead to better access to resources. A researcher has suggested self-efficacy expectations as one factor that accounts for educational differences in responses to outcome measures [22]. However, several studies showed no relationship between self-efficacy and educational levels [23,24]. Most studies on the effects of economic levels on self-efficacy showed no significant difference [23,24].

Understanding the self-efficacy of family caregivers can assist medical teams to understand their capability belief in taking care of patients with oral cancer at home, identify relevant influential factors, and provide countermeasures to enhance their capability belief in patient care. This may improve the home care quality for patients with oral cancer. Therefore, the purpose of this study was to assess the self-efficacy of the primary family caregivers of patients with oral cancer at home.

## 2. Methods

### 2.1. Study Design

The current study adopted a cross-sectional descriptive research design and convenience recruiting to facilitate the sampling and discussion on the self-efficacy of the primary family caregivers of patients with oral cancer at home.

### 2.2. Sample and Procedure

In total, 107 primary family caregivers of outpatients were recruited for a structured questionnaire survey. The participants were enrolled from the radiology outpatient department of a teaching hospital in northern Taiwan from May 2016 to May 2018. Only patients who (1) were aged ≥20 years; (2) were diagnosed as having oral cancer; and (3) received oral cancer-related surgery, chemotherapy, or radiotherapy were included. Moreover, the family caregivers of these patients were required to be (1) aged ≥20 years, (2) recognized as the primary family caregivers by the patients, and (3) living with the patients.

After this study passed the ethical review and the family caregivers signed the informed consent form, a research assistant distributed our questionnaires to the family caregivers. The assistant checked whether the retrieved questionnaires were completely filled out immediately after the caregivers submitted them. The participants who missed items were asked to fill them out. Regarding patient medical characteristics, they were all collected from medical records by the research assistant.

### 2.3. Ethical Considerations

This study was approved by the institutional review board of a teaching hospital in northern Taiwan (VGHIRB No.: 2014-04-001AC). The research assistant verbally explained the research objective, data protection principles, and research procedures to obtain the participants’ consent and asked them to sign the informed consent form. Codes were used in the questionnaire in place of personal information to protect participant privacy. For participants who were unwilling to proceed with the survey or were not physically suitable for further investigations, the research assistant acknowledged their withdrawal intention and stopped collecting their data.

## 3. Measures

### 3.1. Sociodemographic Variables

The current study collected the sociodemographic variables of the family caregivers and patients’ medical characteristics. The collected sociodemographic variables were sex, age, marital status, education level, religious affiliation, employment status, and household income. The collected medical characteristics were the time of sickness, stage of cancer, current treatment status, and treatment side effects. Information related to the family caregivers, such as the family caregivers’ relationships with the patients, manner of care, and care time, were also collected.

### 3.2. Caregiver Caregiving Self-Efficacy Scale-Oral Cancer

The current study applied the Caregiver Caregiving Self-Efficacy Scale-Oral Cancer (CSES-OC) [26] to estimate the self-efficacy of the family caregivers. The scale consisted of 18 items. According to factor analysis, the scale could be divided into four subscales: acquiring resources (AR; six items), managing sudden and uncertain patient conditions (MS; five items), managing patient-related nutritional issues (MN; four items), and exploring and making decisions on patient care (MD; three items). Some examples of the items for AR are “I am confident that I am able to acquire financial support”, “I am confident that I am able to seek consultation on the provision of sick family member care”, and “I am confident that I am able to acquire respite from caregiving”. Examples for MS are “I am confident that I am able to manage the sudden onset of conditions in the sick family member”, “I am confident that I am able to handle uncertainty about cancer progression”, and “I am confident that I am able to handle the sick family member’s uncertainty about death”. Examples for MN are “I am confident that I am able to prepare a suitable diet” and ”I am confident that I am able to improve the sick family member’s willingness to eat”. Examples of the items for MD are “I am confident that I am able to explore the most suitable care for the sick family member” and ” I am confident that I am able to make decisions on sick family member care”. The Cronbach’s alpha of each subscale ranged between 0.78 and 0.91, and that of the overall scale was 0.95. The test–retest reliability with a 2-week interval was *r* = 0.83 (*p* < 0.001), and its criterion-related validity with the General Self-Efficacy Scale was *r* = 0.59 (*p* < 0.001). Regarding the scale used, an 11-point Likert-type scale ranging from 0 (not at all confident) to 10 (completely confident) points was adopted, where the higher the total score, the higher the self-efficacy [26].

### 3.3. Statistical Analysis

The current study used SPSS for Windows (version 22.0; SPSS, Chicago, IL, USA) for the data processing. Descriptive statistics, such as means, SDs, frequencies, and percentages, were obtained to examine the family caregivers’ sociodemographic variables, patients’ medical characteristics, caregiver–patient relationships, manner of care, care times, and caregiving self-efficacies. The differences in the variables in caregiving self-efficacy (e.g., family caregivers’ sociodemographic variables, patients’ medical characteristics, caregiver–patient relationships, and manner of care) were estimated using the independent sample *t*-test and analysis of variance (ANOVA). In addition, a Pearson product–moment correlation test was performed to verify the correlation between caregiver age, care time, patient time of sickness, and caregiving self-efficacy.

## 4. Results

### 4.1. Sociodemographic Variables of the Primary Family Caregivers and the Manner of Care

The current study recruited 107 primary family caregivers as participants, with a mean age of 51 years (SD = 10.8 years, range = 20–70 years). Among the participants, 91.6% were female, 72.9% were the patients’ spouses, 56.1% had an education level of senior high school and above, 87.9% were married, 26.2% were continuing their job, 47.7% had an annual household income of <TWD 500,000, 86.9% had a religious affiliation, and 26.2% had a chronic disease (Table 1). Moreover, 41.1% provided care with the assistance of other caregivers, 40.2% provided care without rest, 83.20% had no experience in patient care, and the mean care time was 36.4 months (SD = 40.3 months, range = 1–171 months; Table 1).

### 4.2. Patients’ Medical Characteristics

Among the 107 patients with oral cancer, the mean time of sickness was 42.5 months (SD = 44.4 months, range = 1–171 months). Of all the patients, 36.4% had stage IV oral cancer, 78.5% had completed their treatment, and 36.4% were still experiencing the side effects of the treatment (Table 2).

### 4.3. Caregiving Self-Efficacy of the Primary Family Caregivers

The CSES-OC was used to measure the self-efficacy of the primary family caregivers. The overall and subscale (i.e., AR, MS, MN, and MD) scores were considered. The mean overall self-efficacy score was 6.87 (SD = 1.65). Moreover, of all the subscales, MN demonstrated the highest mean score of 7.56 (SD = 1.83), followed by MD (7.05, SD = 1.92), AR (6.89, SD = 1.80), and MS (6.17, SD = 2.09) (Table 3).

### 4.4. Differences in the Sociodemographic Variables of the Primary Family Caregivers and Manner of Care in Caregiving Self-Efficacy

No significant correlations were discovered between the overall self-efficacy score and age (*r* = 0.06, *p* > 0.05) and between the overall self-efficacy score and care time (*r* = 0.08, *p* > 0.05). Moreover, no significant differences were noted for the other sociodemographic variables and manner of care in caregiving self-efficacy (Table 1).

### 4.5. Differences in Medical Characteristics in Caregiving Self-Efficacy

No significant correlations were discovered between the time of sickness and the overall self-efficacy score (*r* = 0.11, *p* > 0.05). Moreover, the differences among patients’ other medical characteristics in the overall self-efficacy were nonsignificant (Table 2).

## 5. Discussion

In this study, the researchers analyzed the caregiving self-efficacy of the primary family caregivers of patients with oral cancer. Results of the current study may aid professional caregivers in understanding the capability belief of primary family caregivers in facing challenges during the care process and the most challenging tasks they are likely to encounter.

According to the self-efficacy classification proposed by Kobau and DiIorio [27], a self-efficacy score of 4–7 (range: 0–10) denotes a moderate level of self-efficacy. Here, the mean caregiving self-efficacy score was 6.87, indicating that the caregivers in this study had moderate self-efficacy. However, because the scoring methods used for measuring self-efficacy have varied between previous relevant studies [28,29,30], the researchers could not compare the results of the current study with those of other studies directly. The mean self-efficacy score of the current study was close to that of Liang, Yates, Edwards, and Tsay [22], where the opioid-taking self-efficacy of patients with cancer was estimated, and it was slightly lower than that of Kobau and DiIorio [27], where the self-efficacy of patients with epilepsy was assessed. The possible reason for this was that the care difficulty differed between diseases, which may have affected the participants’ perceived level of capability.

Here, the caregiving self-efficacy in the MN dimension scored the highest, with a mean score of 7.56. Handling the nutritional issues of patients might not be the most challenging task for caregivers. Increasing their willingness to eat and preparing suitable food for them [26] were found to be essential behavior tasks to promote their physiological recovery.

The self-efficacy in the MD dimension scored the second highest, with a mean score of 7.05. In this dimension, the behavior tasks relevant to caregiving self-efficacy included managing the side effects due to cancer treatment and making treatment-related decisions [26]. These types of behavior tasks aim at providing home-based medical assistance.

Moreover, the AR dimension scored the third highest, with a mean score of 6.89. Here, the caregiving self-efficacy-related behavior tasks encompass managing emotional issues, receiving care counseling, and being able to rest during the care process [26]. Emotional management was related to tasks such as dealing with the emotions of patients who were facing oral cancer treatment and prognosis, as well as the emotions of caregivers themselves [16,26]. According to the current results, this was the second most challenging set of behavioral tasks. It was a self-assistance behavior task related to the maintenance of the physical and mental health of the caregivers themselves.

Finally, the MS dimension scored the lowest, with a mean score of 6.17. For caregivers, handling the safety and death issues of patients was the most challenging task. The caregiving self-efficacy-related behavior tasks include handling sudden situations, managing the uncertainty in the disease process, and managing poor prognosis [26]. These most difficult care tasks indicate the care priorities for patients and their family caregivers for health care professionals.

Family caregivers’ capability belief (i.e., self-efficacy) is a key factor that affects subsequent care behavior and care results [31,32]. Professional medical personnel can increase family caregivers’ capability belief according to the four sources of efficacy beliefs in the self-efficacy theory: family caregivers’ performance accomplishment, vicarious experience, professional caregivers’ verbal persuasion, and consideration of family caregivers’ physical and emotional arousal [17,32]. Furthermore, professional medical personnel could integrate relevant educational strategies, including diary logs [33], videos, and brochures [32], to improve family caregivers’ capability beliefs in taking care of patients with oral cancer.

In this study, the researchers adopted a cross-sectional descriptive research design. Therefore, the current study could not obtain the changes in family caregiving self-efficacy with respect to the patient’s condition or required care time. The present study involved all patients in the disease period. The timing of patient enrollment was not controlled. Some patients were still undergoing their course of treatment, some patients had finished their treatment. Different times or stages of treatment may affect the challenge of the care of family and, therefore, may affect their ability cognition. In addition, the sample size was small for all sociodemographic and medical variable groups. It is unlikely that statistical differences could be detected in this population. On the other hand, the current research used convenience sampling, which may have caused sampling deviation. Families with large care loads may have been eliminated naturally. The samples were collected from a teaching hospital in northern Taiwan alone, which might affect the inference of the current results.

## 6. Conclusions

Our current results indicated that family caregiving self-efficacy scores in the CSES-OC MS and AR items were the lowest and the second lowest, respectively. The current study recommends that professional medical teams focus their educational strategies and caregiver self-efficacy enhancement strategies on the dimensions that scored relatively low (i.e., handling patients’ safety and death issues and managing physical and mental health problems through self-assistance). For example, issues in these dimensions include managing the emotional distress of a sick family member and the caregiver themself, handling uncertainty about the sick family member’s cancer progression and death, and managing the sudden onset of conditions in the sick family member. Through family caregivers’ performance accomplishment, vicarious experience, professional caregivers’ verbal persuasion, consideration of caregivers’ physical and emotional arousal, and using educational media, the self-efficacy of family caregivers regarding taking care of a patient with cancer may be increased. The current results are from an exploratory study. The cut-off point of the self-efficacy score in this study refers to the research results of other patient groups. The current study suggests that more studies are needed.

## Figures and Tables

**Table 1:** Sociodemographic variables of the primary family caregivers and the differences in overall caregiving self-efficacy between or among groups (*n* = 107).

Variable	Number	%	Mean	SD	*t*/*F*	*p*
Sex					*t* = −0.19	0.85
Male	9	8.4	6.77	1.40		
Female	98	91.6	6.87	1.68		
Relationship with the patient					*t* = −0.37	0.71
Spouse	78	72.9	6.83	1.76		
Other	29	27.1	6.96	1.37		
Education level					*t* = 1.52	0.13
Junior high school and below	47	43.9	7.14	1.66		
Senior high school and above	60	56.1	6.65	1.63		
Marital status					*t* = 0.66	0.51
Married/cohabiting	94	87.9	6.90	1.68		
Other	13	12.1	6.58	1.45		
Employment status					*F =* 1.25	0.30
Resigned	19	17.8	6.33	1.53		
Employed, providing care after work	28	26.2	7.05	1.52		
Employed, on leave	11	10.3	6.45	1.72		
Other	49	45.8	7.06	1.74		
Annual household income (in TWD)					*F =* 0.13	0.88
<500,000	51	47.7	6.80	1.59		
510,000–1,000,000	39	36.4	6.88	1.79		
>1,010,000	17	15.9	7.03	1.61		
Religious affiliation					*t* = 1.32	0.19
Yes	93	86.9	6.95	1.63		
No	14	13.1	6.32	1.79		
Chronic disease					*t* = 0.01	0.99
Yes	28	26.2	6.86	1.78		
No	79	73.8	6.87	1.62		
Manner of care					*F =* 1.16	0.32
With the assistance of other caregivers	44	41.1	7.16	1.56		
Independent, full-day care	28	26.2	6.63	1.93		
Independent, non-full-day care	35	32.7	6.69	1.52		
Weekly care time					*F =* 0.24	0.78
Without rest	43	40.2	6.88	1.77		
With weekly rest time	24	22.4	7.04	1.65		
With irregular rest time	40	37.4	6.74	1.55		
Past care experience					*t* = 1.02	0.31
Yes	18	16.8	6.50	1.48		
No	89	83.2	6.94	1.69		

**Table 2:** Patients’ medical characteristics and the differences in overall caregiving self-efficacy between or among groups (*n* = 107).

Variable	Number	%	Mean	SD	*t*/*F*	*p*
Stage of cancer					*F =* 0.50	0.68
Stage I	22	20.6	7.00	2.09		
Stage II	32	29.9	6.59	1.55		
Stage III	14	13.1	7.16	1.81		
Stage IV	39	36.4	6.91	1.43		
Current treatment status					*t* = 0.79	0.43
Receiving treatment	23	21.5	6.62	1.67		
Not receiving treatment	84	78.5	6.93	1.65		
Side effects					*t* = 0.84	0.40
Yes	39	36.4	6.69	1.60		
No	68	63.6	6.97	1.69		

**Table 3:** Caregiving self-efficacy in primary family caregivers (*n* = 107).

Variable	Mean	SD	Minimum	Maximum
Acquiring resources (AR)	6.89	1.80	1.50	10.00
Managing sudden and uncertain patient conditions (MS)	6.17	2.09	1.00	10.00
Managing patient-related nutritional issues (MN)	7.56	1.83	1.75	10.00
Exploring and making decisions on patient care (MD)	7.05	1.92	2.00	10.00
Total self-efficacy	6.87	1.65	2.00	10.00

## Data Availability

The data presented in this study are available from the corresponding author upon reasonable request.
